# On the Employment of Oil of Turpentine and Opium in Large Doses in the Treatment of Severe Puerperal Diseases

**Published:** 1859-04

**Authors:** E. Bonfils

**Affiliations:** Bulletin General de Therapeutique


*On the employment of Oil of Turpentine and Opium in Large Doses in the Treatment of severe Puerperal Diseases.* By Dr. E. BONFILS. (Bulletin General de Therapeutique, May 20, 1858.)

M. Trousseau has lately employed, with considerable success, a method of treatment, proposed originally by Dr. Graves, in puerperal diseases. This treatment consists in giving opium and oil of turpentine in large doses to women in child-bed who are attacked with metro-ovaritis, peritonitis, uterine phlebitis, &c. Among other cases, M. Trousseau has treated in this manner, and with success, a woman attacked with peritonitis, and double pleuro-pneumonia. He also employed this plan in another case of a woman attacked with general and very severe peritonitis, which was very rapidly checked and afterwards cured; but although the cure appeared to be permanent, the patient was unfortunately seized with hectic symptoms of an insidious character, and sunk under what appeared to be a putrid infection. In the first case the opium was prescribed in pills and the turpentine in injection.. At first five centigrammes (about one grain) of opium were given in five pills, to be taken daily; then the dose was gradually augmented till it reached about two grains a-day. The opium was continued thirteen days. The turpentine was administered at first in doses of ten grammes (about two drachms and a half) in two glysters, one in the morning and the other in the evening; then the quantity was progressively augmented to thirty grammes (about seven drachms and a half). In the second case the opium [illegible]s also given in pills, in the dose of five centigrammes (about one grain) for three days. The oil of turpentine was administered by the mouth in capsules, each containing one gramme (about the fourth of a drachm) of turpentine; six of these capsules were taken every day, and they were continued for six days.—*Medical News.*

